# The complete mitochondrial genome of *Spurilla braziliana* MacFarland 1909 (Nudibranchia, Aeolidiidae)

**DOI:** 10.1080/23802359.2023.2241693

**Published:** 2023-08-09

**Authors:** Hideaki Mizobata, Kentaro Hayashi, Ryo Yonezawa, Andre Lanza, Shigeharu Kinoshita, Kazutoshi Yoshitake, Shuichi Asakawa

**Affiliations:** Department of Aquatic Bioscience, Graduate School of Agricultural and Life Sciences, The University of Tokyo, Tokyo, Japan

**Keywords:** *Spurilla braziliana*, complete mitochondrial genome, nudibranch, sea-slug

## Abstract

*Spurilla braziliana* MacFarland 1909 is a morphologically diverse nudibranch found in the Pacific and Western Atlantic. The complete mitochondrial genome of *S. braziliana* has been constructed using next-generation sequencing technology. The mitochondrial genome is 14,291 bp and contains 13 protein-coding genes, 2 *rRNA* genes, and 23 *tRNA* genes. Molecular phylogenetic analysis using the maximum likelihood method revealed that *S. braziliana* is included in the superfamily Aeolidioidea and forms a monophyletic group with *Berghia stephanieae*, a nudibranch of the family Aeolidiidae. This study reinforces existing taxonomic insights and provides a basis for further molecular phylogenetic analysis.

## Introduction

Members of the genus *Spurilla* are unique nudibranchs capable of storing and utilizing cnidarian nematocysts in their bodies (Greenwood and Mariscal 1984). These sea slugs are widely distributed in the world’s oceans and attract attention from a taxonomic perspective due to their diverse coloration and morphological characteristics (Carmona et al. 2014). Previous research by Carmona et al. (Carmona et al. 2013) has shown that only one species of *Spurilla*, *S. braziliana*, is found in the Pacific Ocean. However, there is significant morphological diversity within *S. braziliana* (Carmona et al. 2014) suggesting that further molecular phylogenetic analysis may be required to distinguish the different morphotypes. This study reports the complete mitochondrial genome sequence of one morphotype of *S. braziliana* from Japan.

## Materials

**Figure 1.**
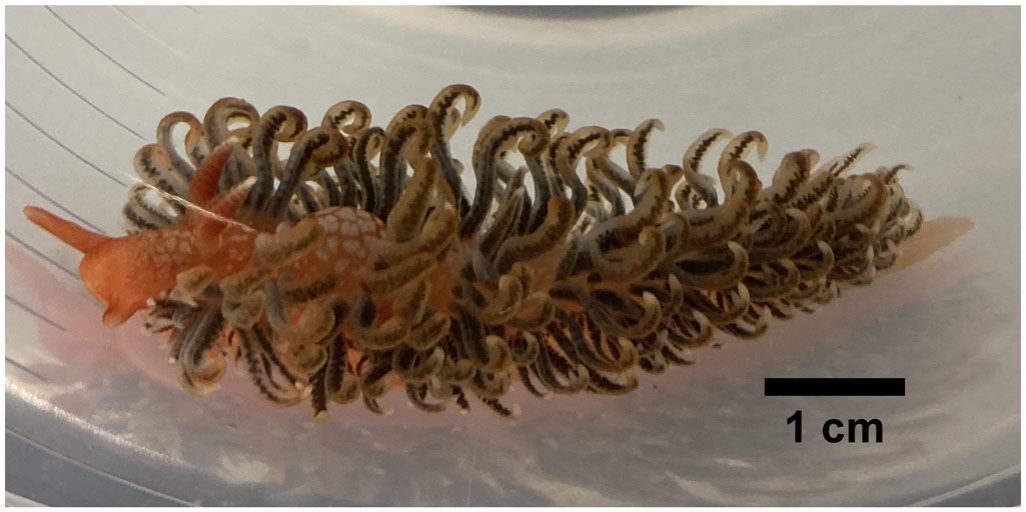
The individual of *S. braziliana* used in this study. Its morphotype is similar to the one shown in Figure 4(E) in the previous taxonomic study (Carmona et al. 2014). The picture was taken by Hideaki Mizobata.

A sample of *Spurilla braziliana* was collected on 10 June 2021 from the intertidal area of Miura City (35°16′18.8′′N 139°34′04.7′′E), Kanagawa Prefecture, Japan (Figure 1) and preserved in NucleoProtect RNA (MACHEREY-NAGEL) for 5.5 months at −80 °C before DNA extraction. The sample was then transferred to 99% ethanol for deposition at the University Museum of the University of Tokyo (http://www.um.u-tokyo.ac.jp/, Assoc. Prof. Takenori Sasaki, sasaki@um.u-tokyo.ac.jp) under voucher number RM34044.

## Methods

Prior to the sequencing process, genomic DNA was extracted from 25 mg of the body wall of the aforementioned sample using the DNeasy Blood & Tissue Kit (QIAGEN, Hilden, Germany) according to the manufacturer’s instructions. A DNAlibrary for PE150 sequencing was prepared with MGIEasy FS DNA Library Prep Set (MGI) and sequenced with DNBSEQ-T7 (MGI) at Genome-Lead Corporation, Japan. A total of 63.6 Gbp of sequence data was obtained from the sequencing, with a Q30 score of 94.08%, indicating sufficient quality for assembly. The reads were assembled with CLC Genomics Workbench version 8.5 (QIAGEN Aarhus A/S) to construct a primary assembly. The primary assembly was polished using bwa mem version 0.7.15-r1140 (Li 2013), samtools version 1.4 (Danecek et al. 2021), and pilon version 1.22 (Walker et al. 2014) with the following commands.

$ bwa index assembly.fasta
$ bwa mem assembly.fasta read1.fastq read2.fastq|samtools view -bS|samtools sort > assembly.fasta.bam
$ samtools index assembly.fasta.bam
$ pilon –genome assembly.fasta –frags assembly.fasta.bam –outdir.

To further improve the polished primary assembly, the sequence was circularized by the ends, cut at a site differing from the circularization point, and polished once more. The raw reads were mapped to the final mitochondrial genome using bwa mem and samtools to ensure proper assembly (Supplementary Figure 1). The average coverage was confirmed to be 7866, further validating the robustness of the assembly and polishing. The polished mitochondrial genome was annotated with MITOS (Donath et al. 2019) and manually corrected using Geneious Prime Java version 11.0.14.1 + 1 (Biomatters Ltd., Auckland, New Zealand). MitoZ (Meng et al. 2019) was used for the accurate annotation of tRNA. The identified *tRNA* genes were initially folded using the RNA-fold web server (Gruber et al. 2008) to generate a rough secondary structure, which was then manually adjusted using VARNA (Darty et al. 2009). Annotations and other mitochondrial genome structures were visualized using OGDRAW (Greiner et al. 2019).

To confirm the species identity of our sample as *S. braziliana*, we performed nucleotide BLAST (blastn) analyses using the web interface of the National Center for Biotechnology Information (NCBI). The *COX1* and *16S rRNA* gene sequences from our specimen were used as queries, with the non-redundant nucleotide collection (nt) database chosen for the search. Default parameters were used for all other settings.

The 13 protein-coding genes within the mitochondrial genome were each individually aligned with their corresponding genes from 22 known Nudibranchia species’ mitochondrial genomes (Table 1) using MAFFT version 7.508 (Katoh and Standley 2013). This was done based on their amino acid sequences, as illustrated in Supplementary Data 1. For each alignment, seven species belonging to the suborder Cladobranchia were selected. The selected 13 alignments were then concatenated. Based on the concatenated alignment, the molecular phylogenetic tree was generated with the maximum likelihood method with 1000 bootstrap replicates in MEGA version 10.1.8 (Kumar et al. 2018).

**Table 1.** The list of species, GenBank accession ID, and references used for alignments (Supplementary Data 1).

Scientific name	GenBank accession ID	Reference
*Spurilla braziliana	LC759638	This study
*Goniobranchus leopardus*	MZ747465	–
*Chromodoris orientalis*	MH550543	Yu et al. (2018)
*Glossodoris acosti*	MZ823409	–
*Hypselodoris festiva*	KU365323	Karagozlu et al. (2016a, 2016b)
*Verconia nivalis*	OL800586	Do et al. (2022)
*Polycera hedgpethi*	MZ713367	–
*Triopha modesta*	MW387958	Do et al. (2022)
*Doris odhneri*	OL800585	Do et al. (2022)
*Asteronotus hepaticus*	MW559976	–
*Carminodoris armata*	OL800584	Do et al. (2022)
*Notodoris gardineri*	DQ991934	Medina et al. (2011)
*Phyllidiopsis krempfi*	MT726194	Kim et al. (2021)
*Phyllidiella pustulosa*	MK279705	Dinh Do, Choi, et al. (2019); Dinh Do, Kim, et al. (2019)
*Roboastra europaea*	AY083457	Grande et al. (2004)
*Phestilla sp.*	PN553001	–
*Dermatobranchus otome	MT527185	Do et al. (2020)
*Tritonia diomedea	KP764765	Sevigny et al. (2015)
*Melibe leonina	KP764764	Sevigny et al. (2015)
*Sakuraeolis japonica	KX610997	Karagozlu et al. (2016a, 2016b)
*Hermissenda emurai	MK279704	Dinh Do, Choi, et al. (2019); Dinh Do, Kim, et al. (2019)
*Berghia stephanieae	MW027646	Melo Clavijo et al. (2021)

Species used to create the molecular phylogenetic tree (Figure 3) are indicated by an asterisk.

## Results and discussion

**Figure 2.**
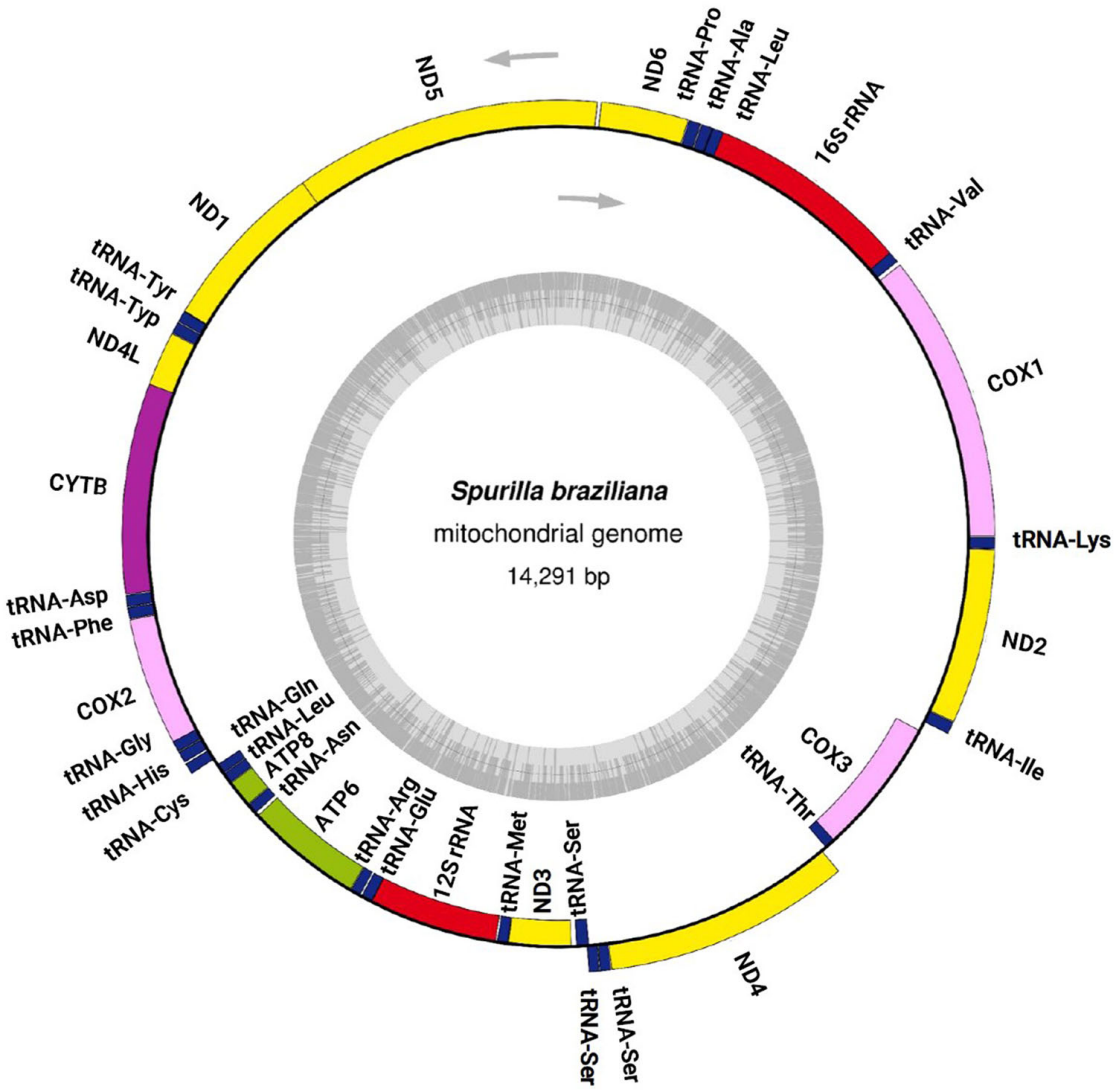
Circular map of the complete mitochondrial genome annotated to show the locations and orientation of its 13 protein-coding genes, 2 *rRNA* genes, and 23 *tRNA* genes. The upper arrows indicate the direction of transcription, while the inner gray circle represents the GC content.

The mitochondrial genome of *S. braziliana* (GenBank Accession ID: LC759638) is 14,291 bp and has a GC content of 33.94%. It consists of 13 protein-coding genes, two *rRNA* genes, and 23 *tRNA* genes (Figure 2). All 13 protein-coding genes exhibit high amino acid sequence similarity with those of other nudibranch species (Supplementary Data 1), suggesting the accuracy of the genome annotation. The gene order is identical to that of *Sakuraeolis japonica* (Karagozlu et al. 2016a, 2016b) and *Hermissenda emurai* (Dinh Do, Choi, et al. 2019; Dinh Do, Kim, et al. 2019), both of which belong to the superfamily Aeolidioidea, the same as *S. braziliana*. The start codons for *S. braziliana* were compared with those of 22 reference species (with 21 species for *ND5* gene). This comparison revealed six different start codons in 23 nudibranchs: ATA, ATT, ATG, ATC, TTG, and GTG. The start codon usage in *S. braziliana* was found to be distinct, particularly the use of GTG in *ATP6*, *ATP8*, *COX1*, and *COX2* genes, a pattern rarely observed in the comparison species (*ATP6*: 1/23 species, *ATP8*: 1/23, *COX1*: 6/23, and *COX2*: 1/23). Within the NADH dehydrogenase gene series, TTG serves as the start codon in 5 genes, excluding *ND4* and *ND5*. For the remaining 4 genes, *COX3*, *CYTB*, and *ND4* adopt ATG as their start codon, while *ND5* employs ATA. *Berghia stephanieae*, which belongs to the same family, Aeolidiidae, as *S. braziliana*, has been reported to exhibit *tRNA-Ser 1* gene duplication (Melo Clavijo et al. 2021). In our *S. braziliana* sample as well, gene duplications of *tRNA-Ser* were observed, and these tRNAs exhibited a unique secondary structure with a missing D-arm (Supplementary Data 2).

Through our blastn analyses of the annotated *COX1* and *16S rRNA* genes, we found that they only exhibited over 90% similarity with two species, *S. braziliana* and *S. neapolitana*, thus confirming that our sample belongs to the genus *Spurilla*. The *COX1* gene showed 100% similarity with one of the *S. braziliana* references, but did not demonstrate complete similarity with the *S. neapolitana* reference. Given that all *Spurilla* nudibranchs found in the Pacific are *S. braziliana* (Carmona et al. 2013), we can confidently assert that our specimen is *S. braziliana*.

**Figure 3.**
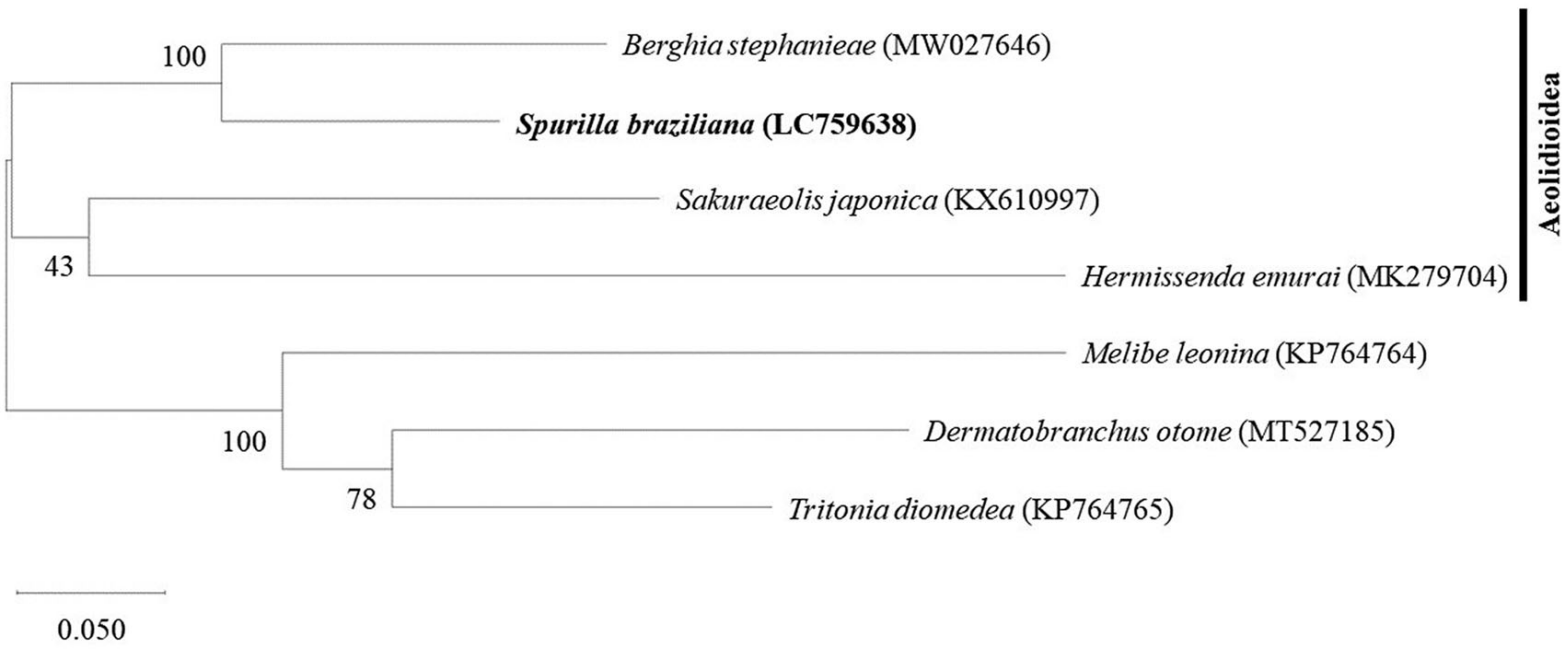
Maximum likelihood phylogenetic tree of *S. braziliana* and six other Cladobranchia species based on the concatenated amino acid alignments of the 13 protein-coding genes in the mitochondrial genome. Bootstrap support values are shown for each node, based on 1000 replicates. The list of species and their GenBank accession IDs are shown in Table 1.

As shown in the molecular phylogenetic tree (Figure 3), *S. braziliana* forms a monophyletic group with *B. stephanieae* and members of the superfamily Aeolidioidea.

The previous molecular phylogenetic study of the superfamily Aeolidioidea using *COI*, *16S rRNA*, and *H3* genes showed that the monophyletic group containing the genera *Spurilla* and *Berghia* was a sister group to the group containing *Sakuraeolis* (Carmona et al. 2011). The phylogenetic tree generated using all the protein-coding genes of the mitochondrial genome matched these findings. The mitochondrial genome of *S. braziliana*, the first mitochondrial genome of its genus, will provide important data for future taxonomic studies on *Spurilla* nudibranchs, which are known for their highly variable morphology.

## Ethical approval

The ‘Manual for Animal Experiment of the University of Tokyo’ provides ethical guidelines only for mammals, birds, and reptiles, and does not require ethical review for the invertebrates used in this study. Therefore, any specific permission from the ‘Office for Life Science Research Ethics and Safety’ of the University of Tokyo is not necessary for this research.

## Supplementary Material

Supplemental Material

Supplemental Material

Supplemental Material

Supplemental Material

## Data Availability

The genome sequence data that support the findings of this study are openly available in GenBank of NCBI at https://www.ncbi.nlm.nih.gov under the accession ID: LC759638. The associated BioProject, SRA, and Bio-sample numbers are PRJDB15401, DRR450785, and SAMD00585630, respectively.
