# Supplementary figures and images for: The complete mitochondrial genome of Spurilla braziliana MacFarland 1909 (Nudibranchia, Aeolidiidae)
Source: Mitochondrial DNA B Resour. 2023 Aug 9;8(8):862–6. doi: 10.1080/23802359.2023.2241693 (PMC10413916; doi:10.1080/23802359.2023.2241693)

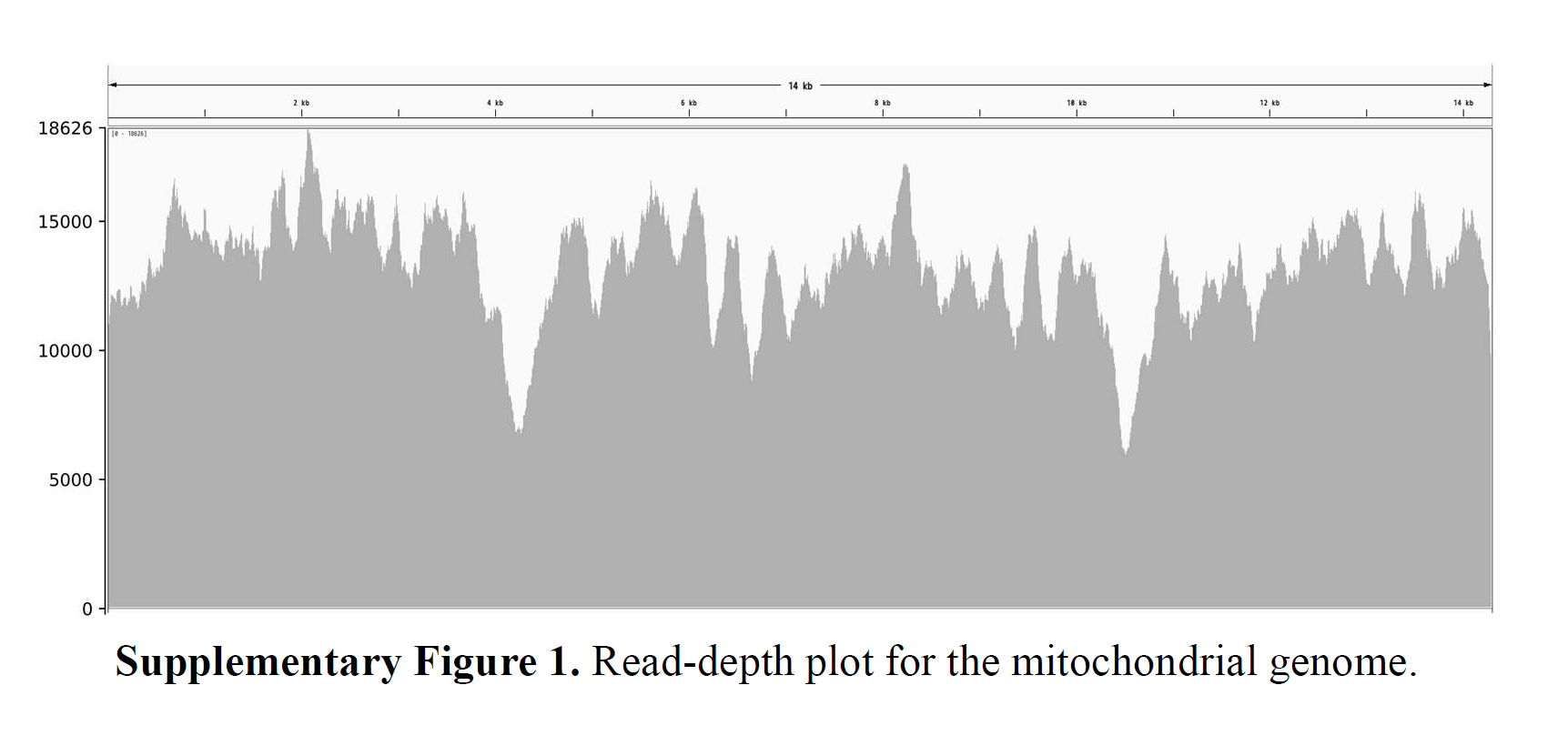

Supplement: Supplemental Material [file TMDN_A_2241693_SM6664.png]

## Slide 1
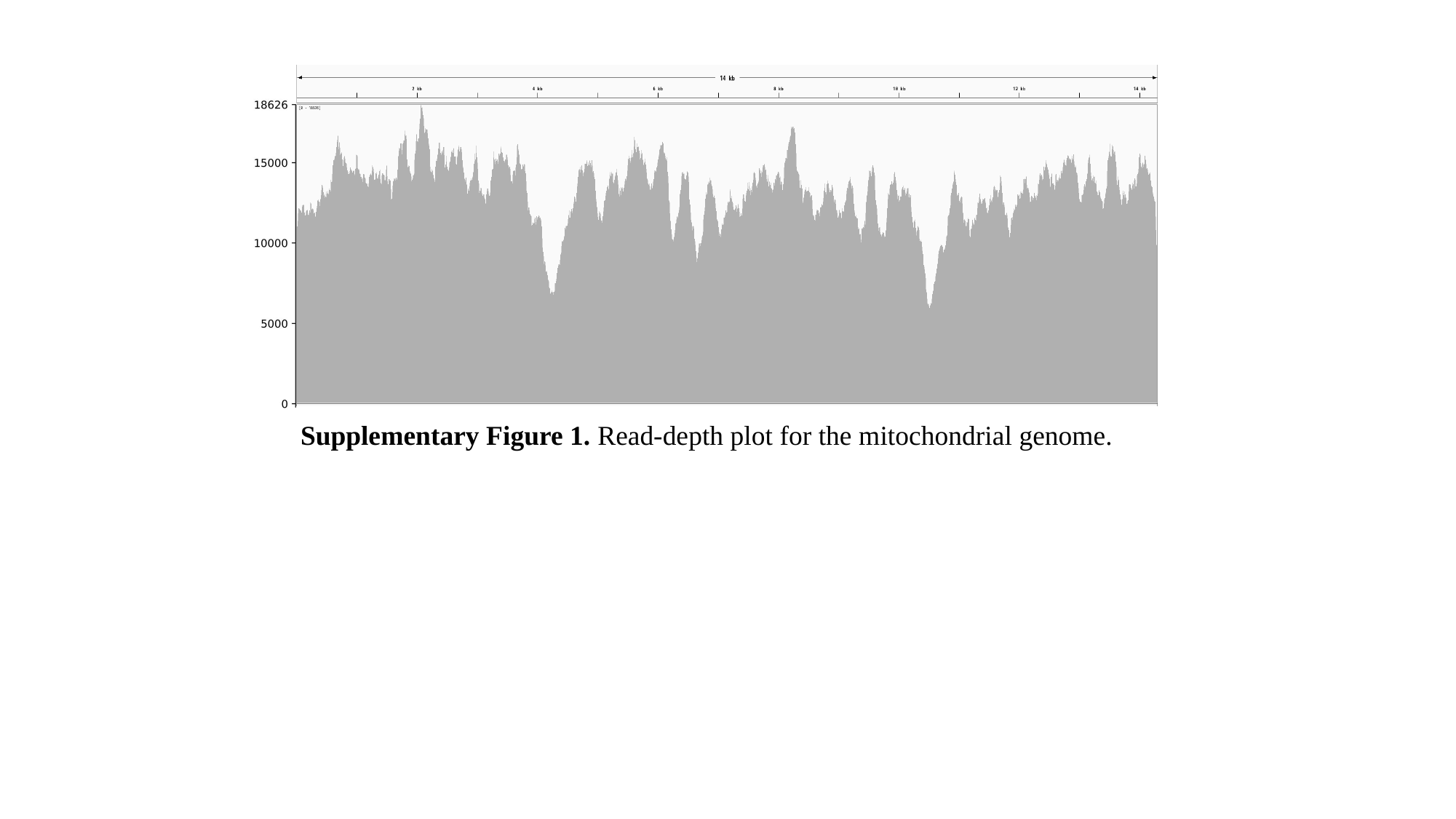

Supplementary Figure 1. Read-depth plot for the mitochondrial genome.

Supplement: Supplemental Material [file TMDN_A_2241693_SM6662.pptx]
