# Supplementary material for: The complete mitochondrial genome of Spurilla braziliana MacFarland 1909 (Nudibranchia, Aeolidiidae)
Source: Mitochondrial DNA B Resour. 2023 Aug 9;8(8):862–6. doi: 10.1080/23802359.2023.2241693 (PMC10413916; doi:10.1080/23802359.2023.2241693)
Supplement: Supplemental Material [file TMDN_A_2241693_SM6658.pptx]

## Slide 1
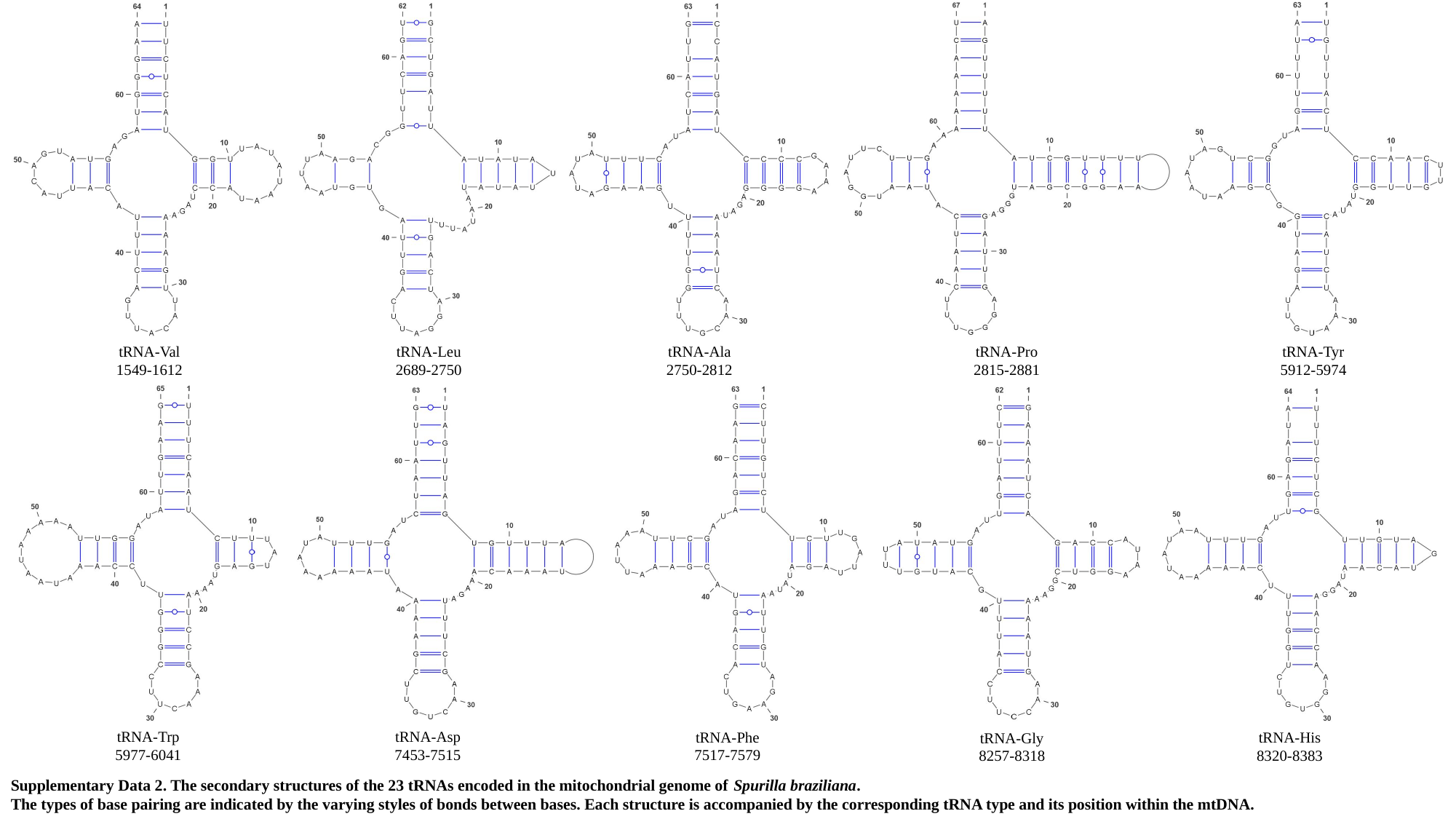

tRNA-Pro
2815-2881
tRNA-Tyr
5912-5974
tRNA-Ala
2750-2812
tRNA-Val
1549-1612
tRNA-Leu
2689-2750
tRNA-Trp
5977-6041
tRNA-Asp
7453-7515
tRNA-Phe
7517-7579
tRNA-His
8320-8383
tRNA-Gly
8257-8318
Supplementary Data 2. The secondary structures of the 23 tRNAs encoded in the mitochondrial genome of Spurilla braziliana.
The types of base pairing are indicated by the varying styles of bonds between bases. Each structure is accompanied by the corresponding tRNA type and its position within the mtDNA.

## Slide 2
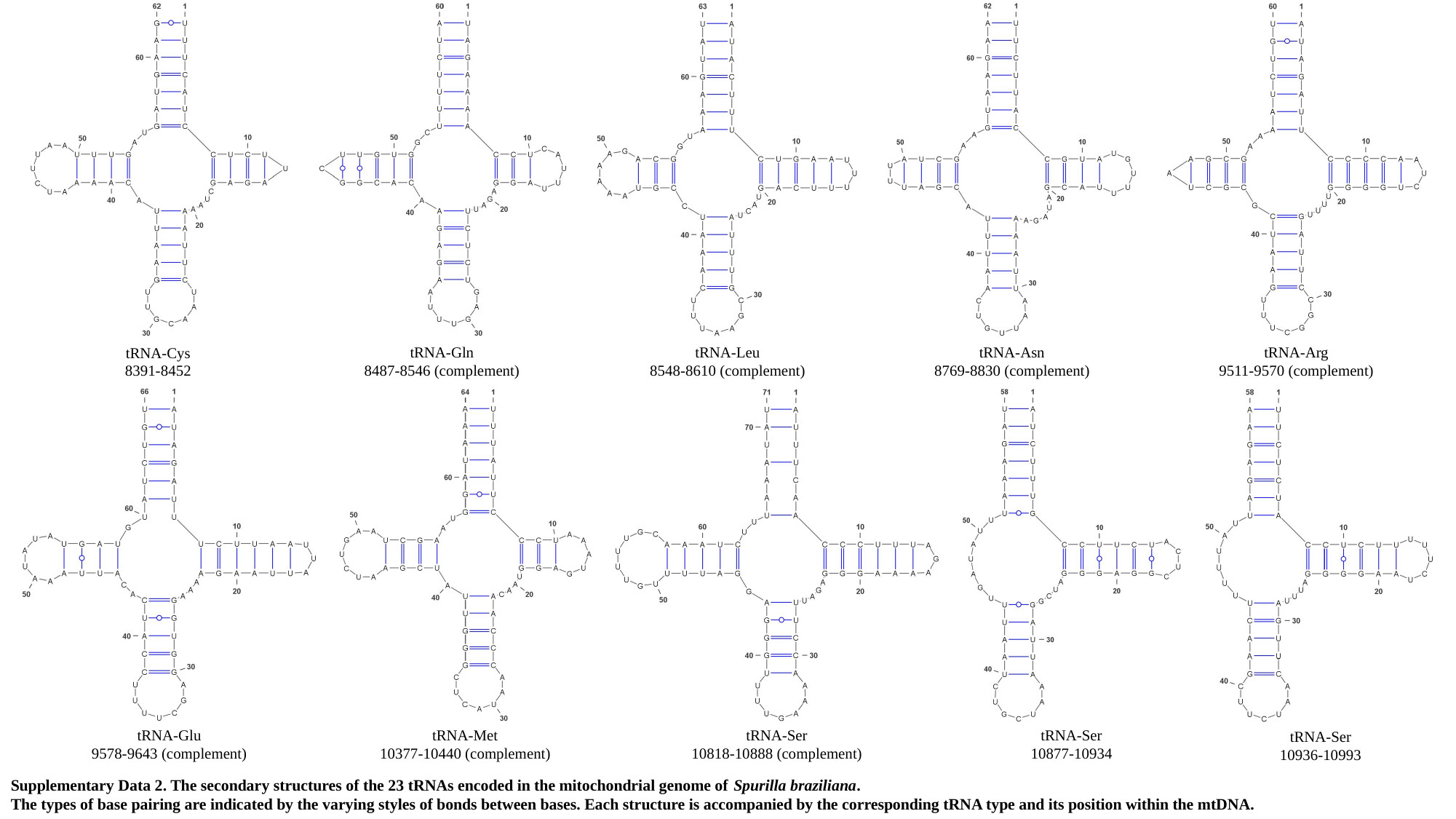

tRNA-Gln
8487-8546 (complement)
tRNA-Leu
8548-8610 (complement)
tRNA-Asn
8769-8830 (complement)
tRNA-Arg
9511-9570 (complement)
tRNA-Cys
8391-8452
tRNA-Glu
9578-9643 (complement)
tRNA-Ser
10818-10888 (complement)
tRNA-Ser
10877-10934
tRNA-Met
10377-10440 (complement)
tRNA-Ser
10936-10993
Supplementary Data 2. The secondary structures of the 23 tRNAs encoded in the mitochondrial genome of Spurilla braziliana.
The types of base pairing are indicated by the varying styles of bonds between bases. Each structure is accompanied by the corresponding tRNA type and its position within the mtDNA.

## Slide 3
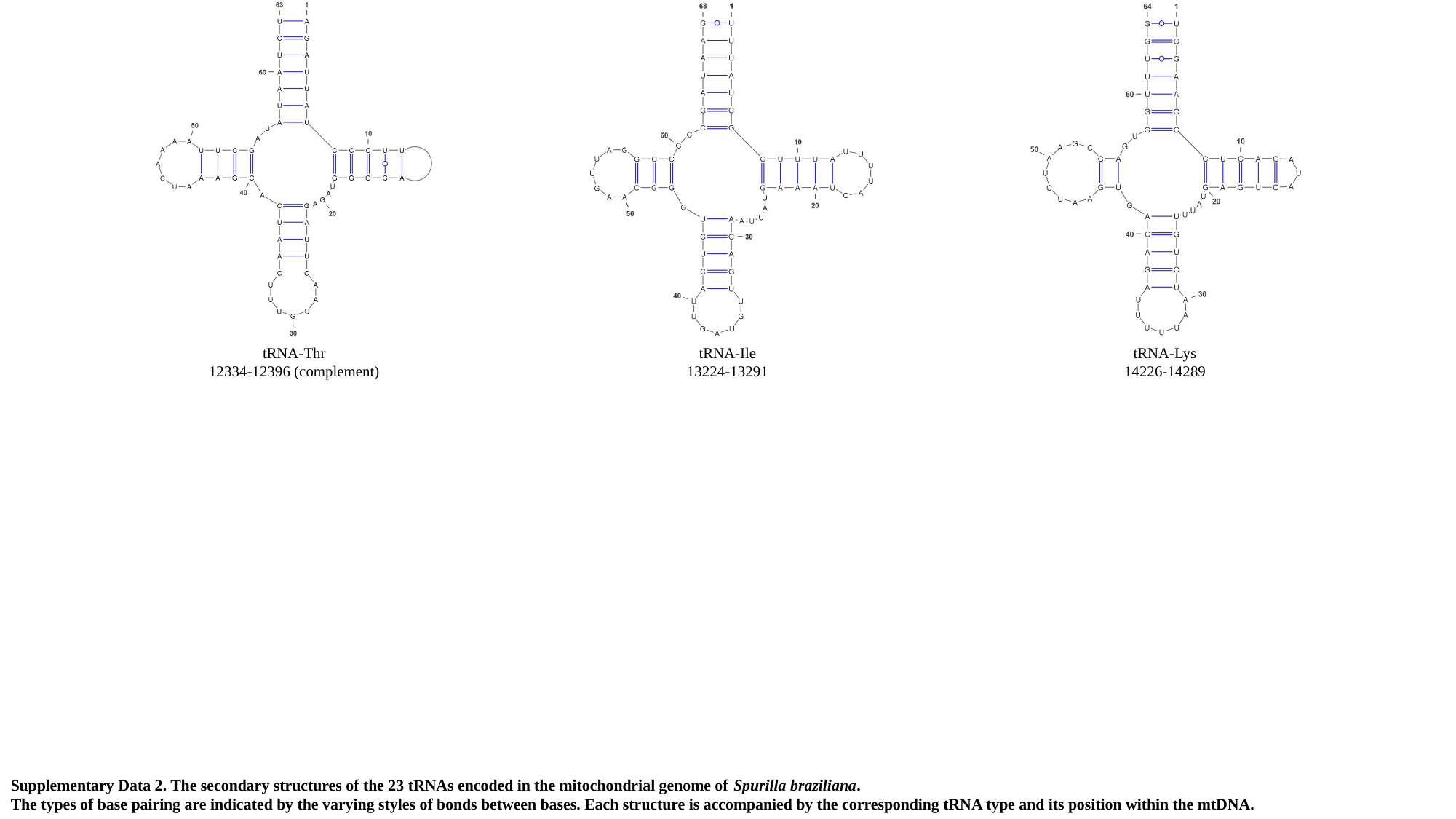

tRNA-Thr
12334-12396 (complement)
tRNA-Ile
13224-13291
tRNA-Lys
14226-14289
Supplementary Data 2. The secondary structures of the 23 tRNAs encoded in the mitochondrial genome of Spurilla braziliana.
The types of base pairing are indicated by the varying styles of bonds between bases. Each structure is accompanied by the corresponding tRNA type and its position within the mtDNA.
