# Supplementary material for: The complete mitochondrial genome of Spurilla braziliana MacFarland 1909 (Nudibranchia, Aeolidiidae)
Source: Mitochondrial DNA B Resour. 2023 Aug 9;8(8):862–6. doi: 10.1080/23802359.2023.2241693 (PMC10413916; doi:10.1080/23802359.2023.2241693)
Supplement: Supplemental Material [file TMDN_A_2241693_SM6649.pptx]

## Slide 1
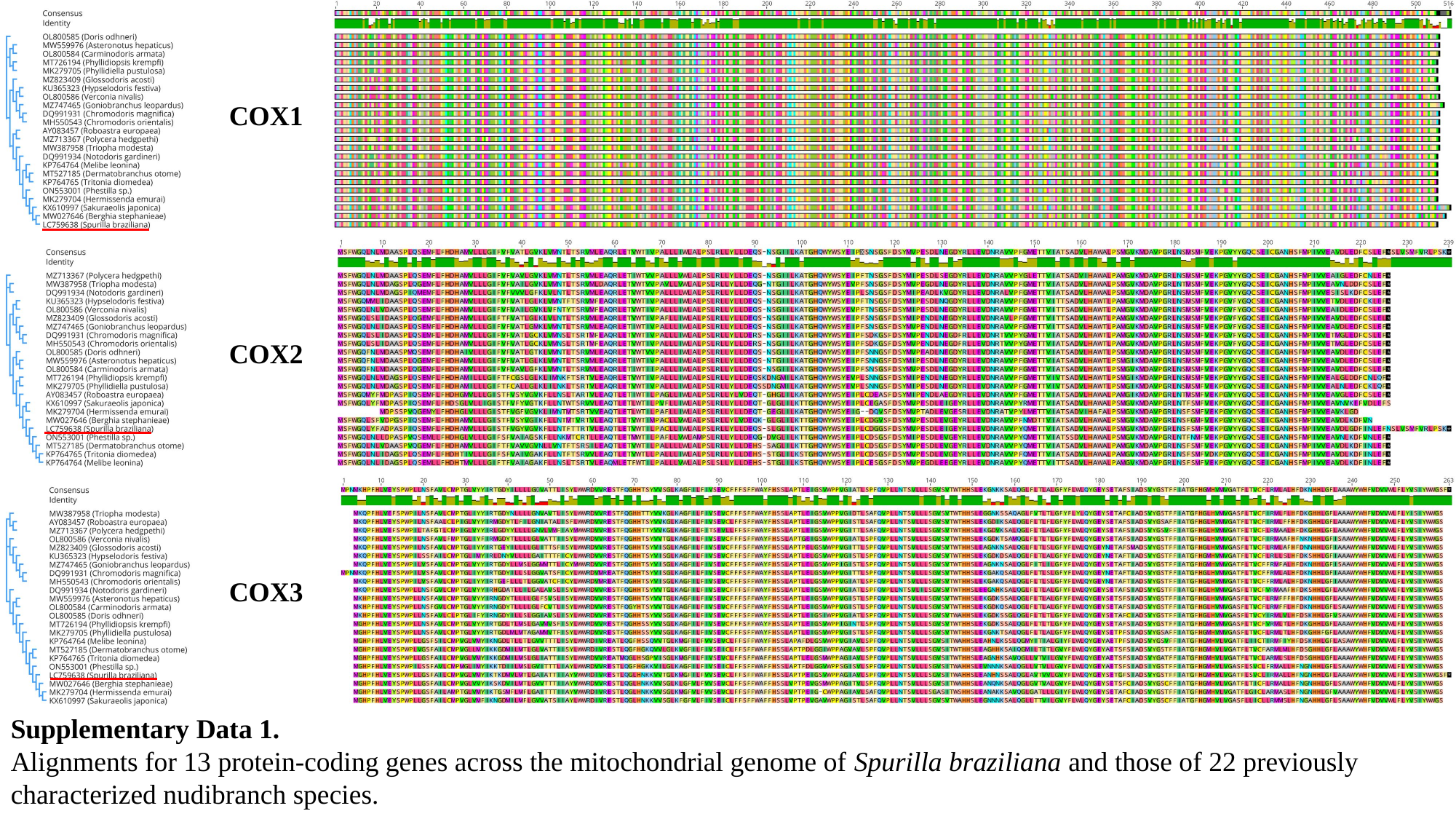

COX1
COX2
COX3
Supplementary Data 1.
Alignments for 13 protein-coding genes across the mitochondrial genome of Spurilla braziliana and those of 22 previously characterized nudibranch species.

## Slide 2
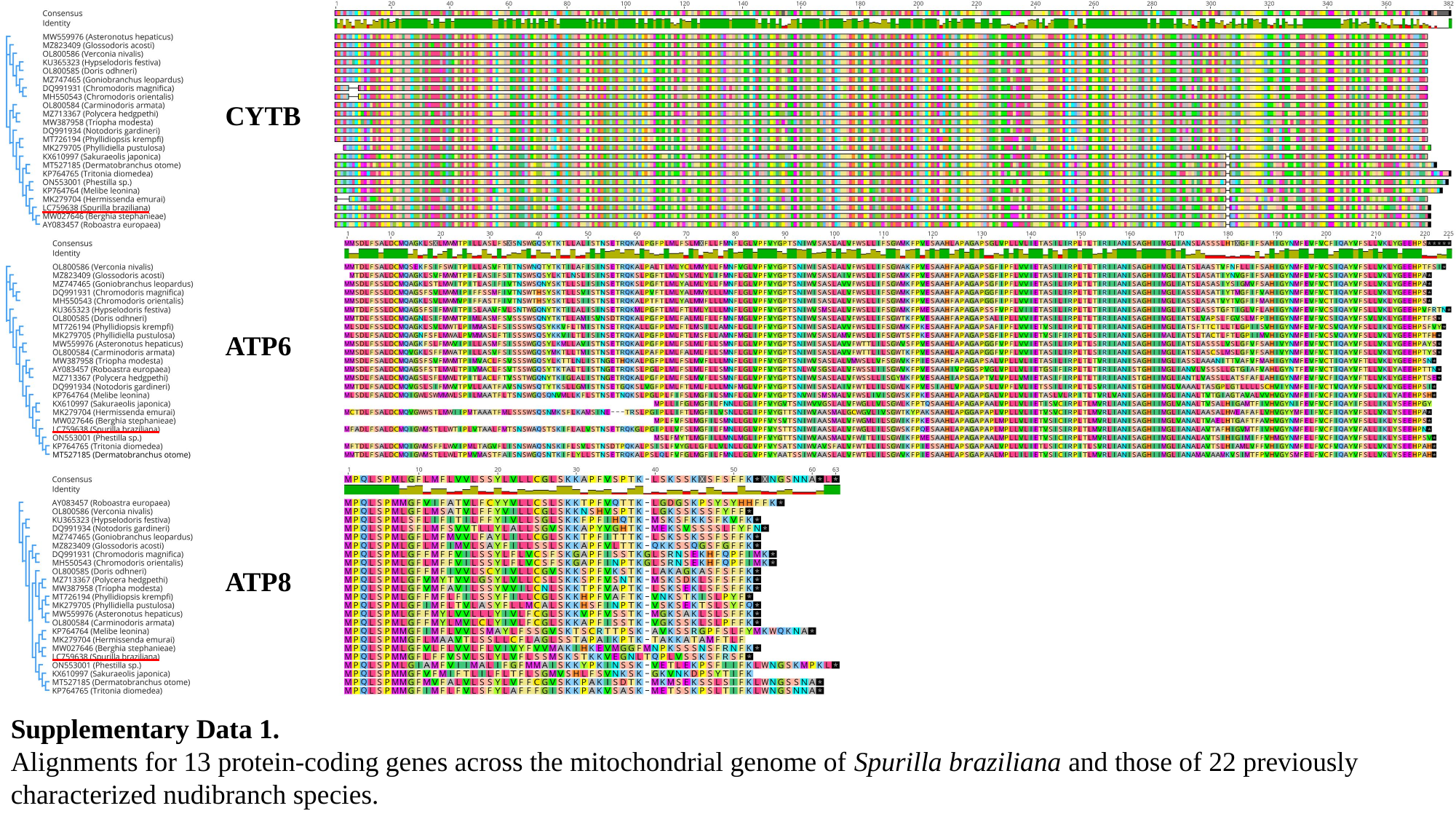

CYTB
ATP6
ATP8
Supplementary Data 1.
Alignments for 13 protein-coding genes across the mitochondrial genome of Spurilla braziliana and those of 22 previously characterized nudibranch species.

## Slide 3
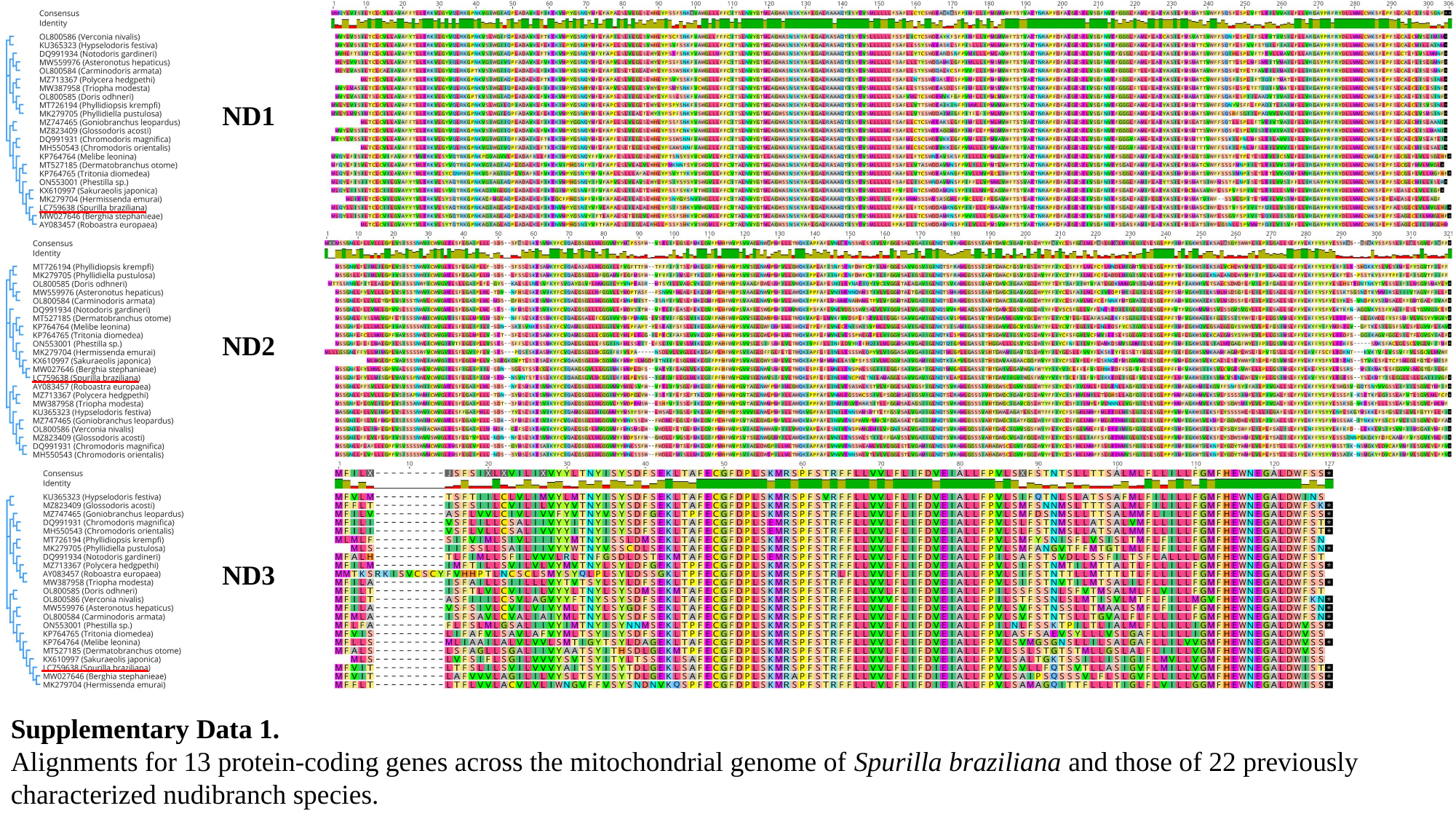

ND1
ND2
ND3
Supplementary Data 1.
Alignments for 13 protein-coding genes across the mitochondrial genome of Spurilla braziliana and those of 22 previously characterized nudibranch species.

## Slide 4
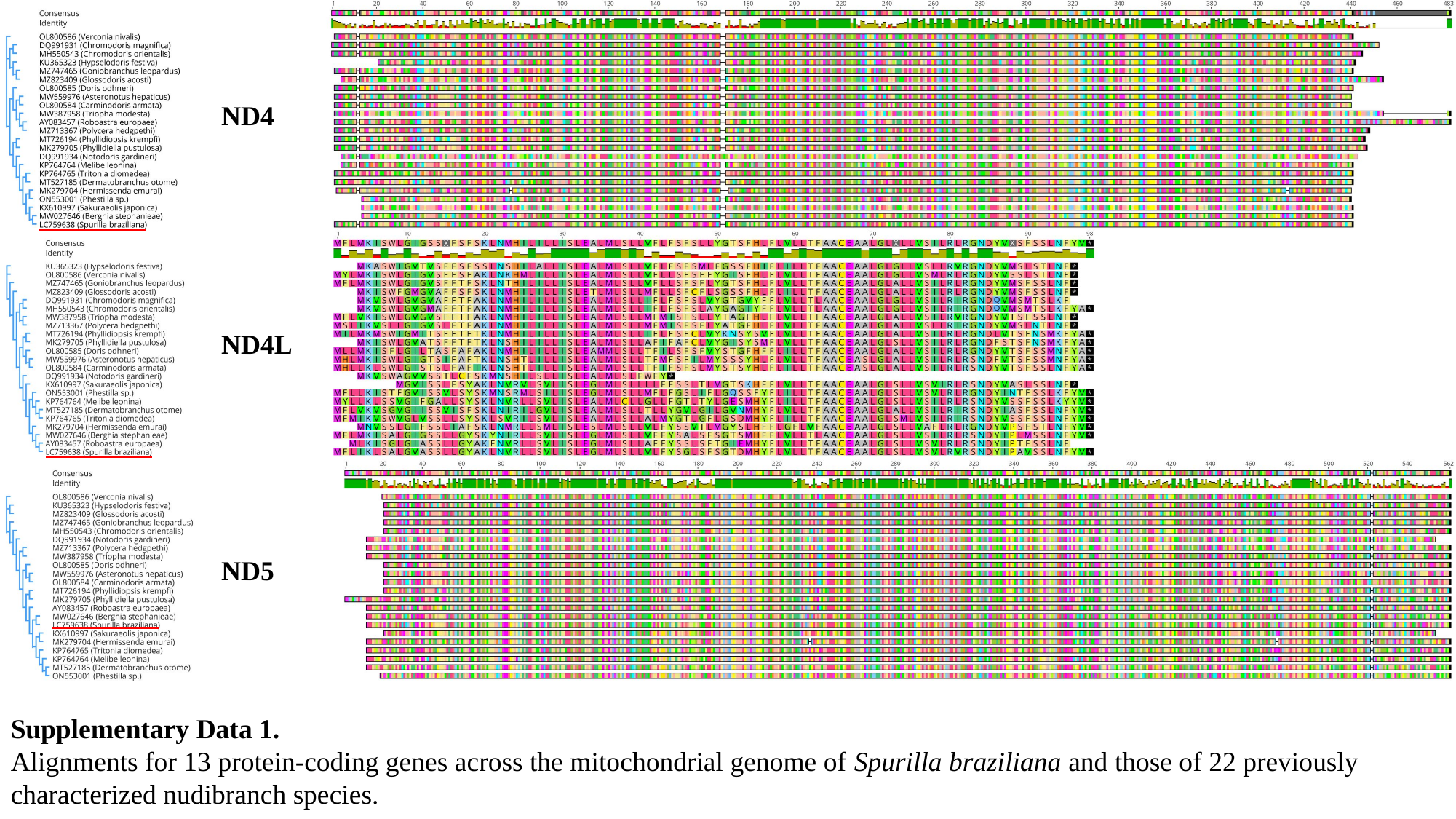

ND4
ND4L
ND5
Supplementary Data 1.
Alignments for 13 protein-coding genes across the mitochondrial genome of Spurilla braziliana and those of 22 previously characterized nudibranch species.

## Slide 5
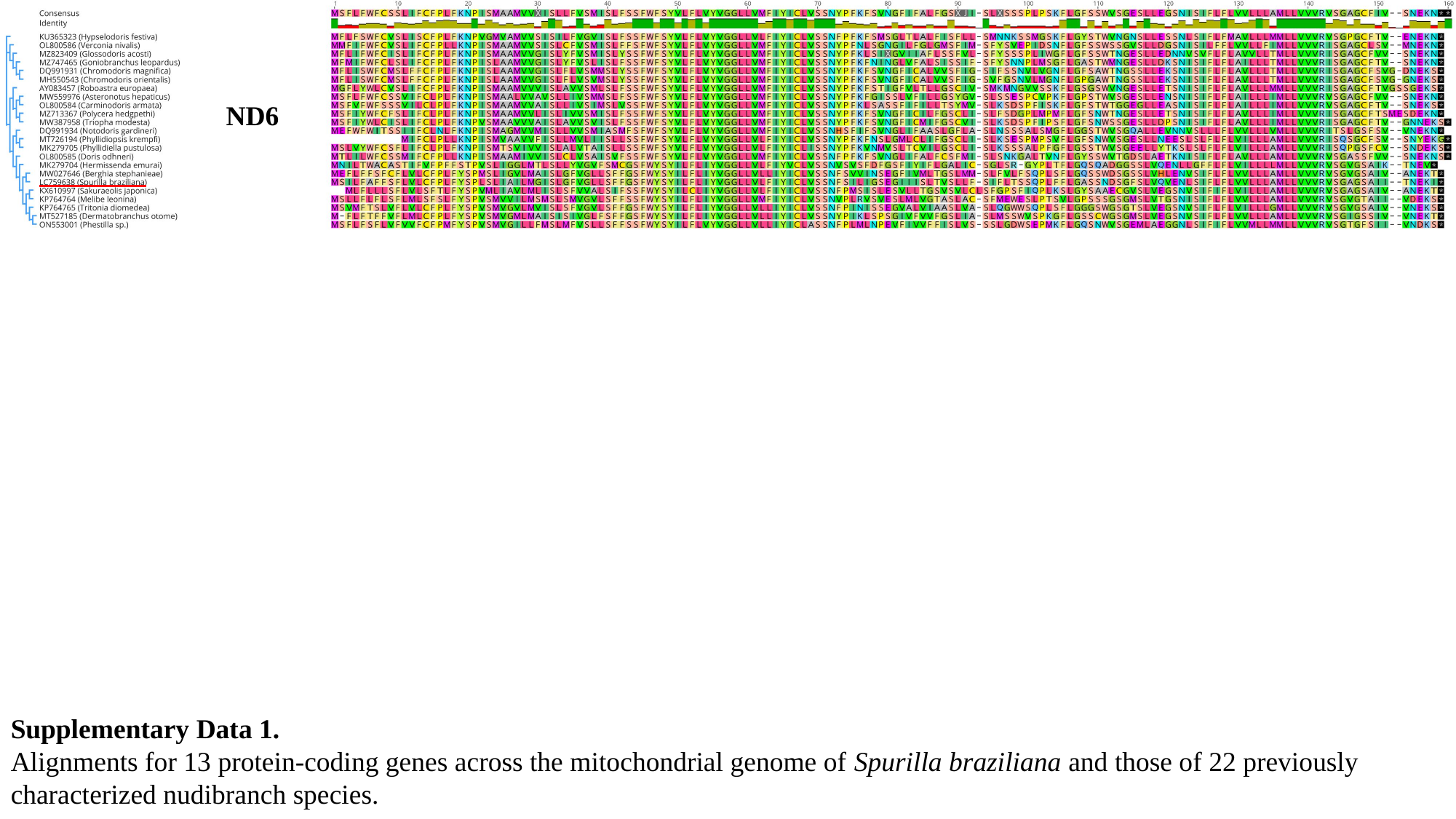

ND6
Supplementary Data 1.
Alignments for 13 protein-coding genes across the mitochondrial genome of Spurilla braziliana and those of 22 previously characterized nudibranch species.
